# Analysis of metabolites in chardonnay dry white wine with various inactive yeasts by ^1^H NMR spectroscopy combined with pattern recognition analysis

**DOI:** 10.1186/s13568-019-0861-y

**Published:** 2019-09-05

**Authors:** Boran Hu, Yang Cao, Jiangyu Zhu, Wenbiao Xu, Wenjuan Wu

**Affiliations:** 1 School of Food Science and Engineering, Yangzhou University, Yangzhou, 225127 Jiangsu China; 2 Department of Medical Oncology, Northern Jiangsu People’s Hospital Affiliated To Yangzhou University, Yangzhou, 225000 Jiangsu, China

**Keywords:** Chardonnay wine, Inactive yeast, Metabolites, Metabolomics, NMR introduction

## Abstract

The study aimed to investigate the effect of five inactive yeasts on the metabolites of Chardonnay dry white wines vinified in 2016 in Shacheng Manor Wine Co. Ltd., Hebei province, China. In this research, proton nuclear magnetic resonance (NMR) spectroscopy coupled multivariate analysis (^1^H NMR-PCA/PLS-DA) were applied to identify and discriminate the different wine products. The results of principle component analysis (PCA) showed that there was significant difference between the metabolites of sample wines with different inactive yeasts, among them, the content of polyols, organic acids, amino acids and choline was notably influenced. The results of partial least squares discrimination analysis (PLS-DA) confirmed that the metabolites contributed to the discrimination of the wines were 2,3-butanediol, ethyl acetate, malic acid, valine, succinic acid, lactic acid, tartaric acid, glycerol, gallic acid, choline, proline, and alanine.

As a commonly seen alcoholic beverage, the consumption of wines has been increasing a lot in recent years. Wine contains a large amount of nutrition, such as ketones, phenols, polyols, amino acids, vitamins, esters, minerals and carbohydrates (Ivanova-Petropulos et al. 2015). With the rapid development of the domestic economy and the gradual improvement of people’s living standards, the consumers have been paying more attention to their health. In this case, wines with good taste, flavor, and a lot of health-care functions are becoming more and more popular in our daily life (Williamson et al. 2012). The quality of the wine usually depends on the physical and chemical indicators, such as the total sugar content, total acid, total solids content, alcohol content, phenol, and pH (Moreno-Arribas and Polo 2005). However, the composition of the wine is very complex, and some trace metabolites can’t be represented by these indicators. In this case, the introduction of wine metabolomic “Fingerprint” is necessary (Nicholson et al. 1999; Serkova and Niemann 2006; Jiménez-Girón et al. 2015). According to the more objective and accurate method, the physiological statues and biological characteristics of different wines can be described completely.

Inactive yeast is a deactivated yeast, a strain of *Saccharomyces cerevisiae*, which is mainly used as a food product. In recent years, inactive yeast has also been used in winemaking industries. Inactive yeast components are complex, including yeast polysaccharides, nucleotides, autolytic yeast, amino acids, multivitamins, minerals, etc. It can play a positive role in wine fermentation (Mekoue Nguela et al. 2015). Inactive yeast can promote rehydration activation of active dry yeast. Pozo-Bayón et al. investigated that inactive dry yeast could increase the rate of rehydration and wine fermentation of active dry yeast (Pozo-Bayón et al. 2009).

Soubeyrand et al. explained that in order to improve the overall fermentation efficiency, inactive dry yeast released its own fragments of cell wall into the system to form micellar particles to reduce the total surface tension of water, while repairing damaged yeast cell membranes (Soubeyrand et al. 2005).

In addition, Chiaramonti et al. (1997) showed that all four inactive dry yeasts could increase the rate of alcohol fermentation when the number of live yeast was 500 mg/L. Furthermore, inactivated yeast can affect the aroma components composition in wine. According to Comuzzo et al. low inactive yeast amounts increased the volatility of some esters, giving more flowery and fruity notes to the wine; higher amounts increased fatty acid content in the wine headspace, producing yeasty, herbaceous and cheese-like smells (Comuzzo et al. 2006).

Currently, the common detection methods mainly include liquid chromatography–mass spectrometry (LC–MS) technique (Huang et al. 2014; Hochberg et al. 2015; Moreno-García et al. 2015), gas chromatography–mass spectrometry (GC–MS) technique (Antalick et al. 2015; Lee et al. 2015), NMR technique (Furlan et al. 2014; Mazzei et al. 2013; Son et al. 2008), etc. Metabolomics based on NMR and LC combined with pattern recognition analysis was used to characterize the difference of metabolites in the wines vinified by various inactive yeasts. It is of great significance to improve the wine-making process and the wine quality. In this research, the proton nuclear magnetic resonance (NMR) spectroscopy coupled multivariate analysis (PCA/PLS-DA) was applied to investigate the effect of five inactive yeasts on the metabolites of Chardonnay dry white wines vinified in 2016 in Shacheng Manor Wine Co. Ltd., Hebei province, China.

## Materials and methods

### Wine sample

Chardonnay grape was produced in Shacheng Manor Wine Co. Ltd., Hebei province, China. The Shacheng has a temperate continental monsoon climate and the average yearly cumulative temperature above 10 °C was 3532 °C. Heat of this production area was sufficient and the hydrothermal coefficient was less than 1.5. The average temperature difference between day and night was 12.5 °C, which was suitable for the accumulation of sugar.

**Fig. 1:**
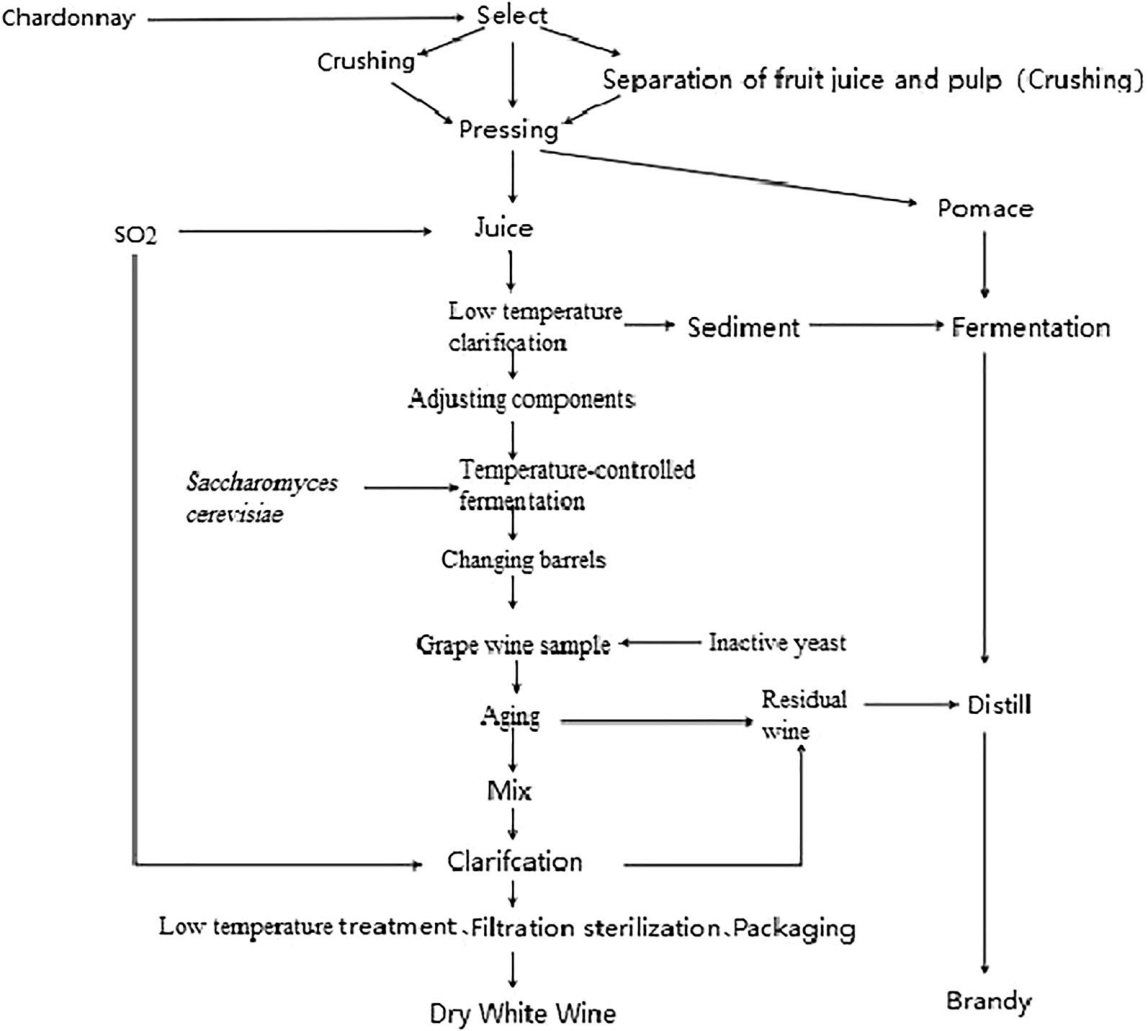
Brewing process of Chardonnay dry white wine with various inactive yeasts

The wine was brewed following the standard process (Fig. 1) in 2016. After de-stemming and crushing the grapes, and then added the yeast to ferment at 18–22 °C for 8–10 days. By gently pressing the pomace and separating the supernatant, the original wine was obtained and then tank-switched. The physical and chemical indexes of the original wines were complied with the Chinese national standard (GB15037-2006). Five inactive yeasts were added in the original wine before wine aging. The original wine was regarded as the control with no addition. The details of the wine samples with various inactive yeasts are shown in Table 1. Samples were stored at − 4 °C for 2 months.

**Table 1:** Information of wine samples with various inactive yeast

Wine samples	Inactive yeast varieties	Dosage (g/L)	Abbreviation	Origin	Breed
1	OptiMUM-White^®^	0.4	OW	Institut Coopératif du Vin, France	*Saccharomyces cerevisiae*
2	Opti-LEES	0.4	OL		
3	Booster Blanc^®^	0.4	BB		
4	Noblesse^®^	0.4	NL		
5	Mannostab^®^	0.4	MS		
6	Control (original wine)	0	C		

### Reagents and equipment

DSS (NMR Biotechnical Co., Ltd., Shanghai, China); D_2_O (Tenglong Weibo Technology Co., Ltd., Qingdao, China); Oxalic acid, Sodium oxalate (Su Yi Chemical Reagent Co., Ltd., Shanghai, China); Ethanol (Su Yi Chemical Reagent Co., Ltd., Shanghai, China).

SNL315SV-230 Freeze dryer (Thermo Co., Ltd., America); AVANCE 600 Nuclear magnetic resonance spectrometer (Bruker Co., Ltd., German); TG16A-WS Desktop high-speed centrifuge (Lu Xiangyi Centrifuge Instrument Co., Ltd., Shanghai, China); BS-224 Electronic balance (Eppendorf Co., Ltd., German); ULT178-6-V49 Ultra low temperature freezer (Revco Co., Ltd., America); RE52-4 Rotary evaporator (Huxi Analysis Instrument Co., Ltd., Shanghai, China); XW-80A Swirl mixer (Huxi Analysis Instrument Co., Ltd., Shanghai, China).

### Pretreatment of wine samples for NMR analysis

Ten milliliters of wine samples were taken, and then centrifuged at the speed of 3000 rpm at 4 °C for 25 min. Three milliliters of supernatant fluid were pre-frozen at − 80 °C for 12 h, and then transferred into the freeze drier for 48 h. Afterward, added 400 μL oxalate buffer (pH 4), 60 μL of 0.5% DSS (internal standard) and 140 μL of D_2_O, and the mixture were centrifuged at 13,000 rpm for 20 min. Took 500 μL of supernatant and loaded them into the 5 mm NMR tube. Each sample was repeated 6 times. NMR experiments were then performed immediately.

### NMR experimental data collection

^1^H NMR spectra of wine samples was collected from AVANCE 600 NMR spectrometer. The temperature of the NMR analysis was set to 298 K. The proton frequency of ^1^H NMR was 600.23 MHz and the spectral width is 7183.9 Hz. The number of sampling points was 32 k. The relaxation delay was set to 2 s and the sampling time was set to 2.3 s. The linewidth enhancement factor was 0.3 Hz. The zgpr pulse sequence was used to suppress the water peak signal, and all the samples were scanned 256 times.

### NMR experimental data processing

After the data collection was completed, the “Fourier transform”, phase adjustment and baseline correction of the spectra were carried out. The assignment and identification of the peak was according to the chemical shift.

The chemical shift interval between δ0 and 10.0 ppm in the NMR spectrum was integrated at the section of 0.005 ppm using Software AMIX. Removed the residual DSS peaks of − 0.5 to 0.5 ppm, 1.74–1.84 ppm and 2.90–2.95 ppm, the residual ethanol peaks of 1.18–1.22 ppm and 3.57–3.72 ppm, and the residual water peaks of 4.8–4.96 ppm. After normalizing the NMR data, imported them into the software SIMCA-P 12.0 for pattern recognition analysis. The peak area of the proton on the given group in the substance was calculated, and the ratio of the peak area to that of the internal standard DSS in one-dimensional ^1^H-NMR spectrum is the metabolite content.

Principal components analysis (PCA) was used to visualize and overview the data, reduce the dimension of the high-dimensional data. In addition, the signal noise could be removed and the discrete trend between samples could be observed. After the PCA, in order to sharpen the separation between observations groups, partial least squares discriminant analysis (PLS-DA) was carried out. By rotating PCA components, we could obtain a maximum separation among classes. Furthermore, PLS-DA could help to distinguish which component carries the class separating information.

## Results

**Fig. 2:**
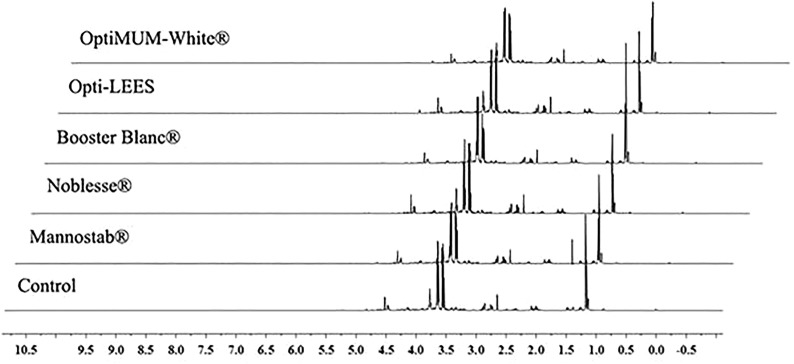
^1^H NMR spectra of Chardonney dry white wine

**Fig. 3:**
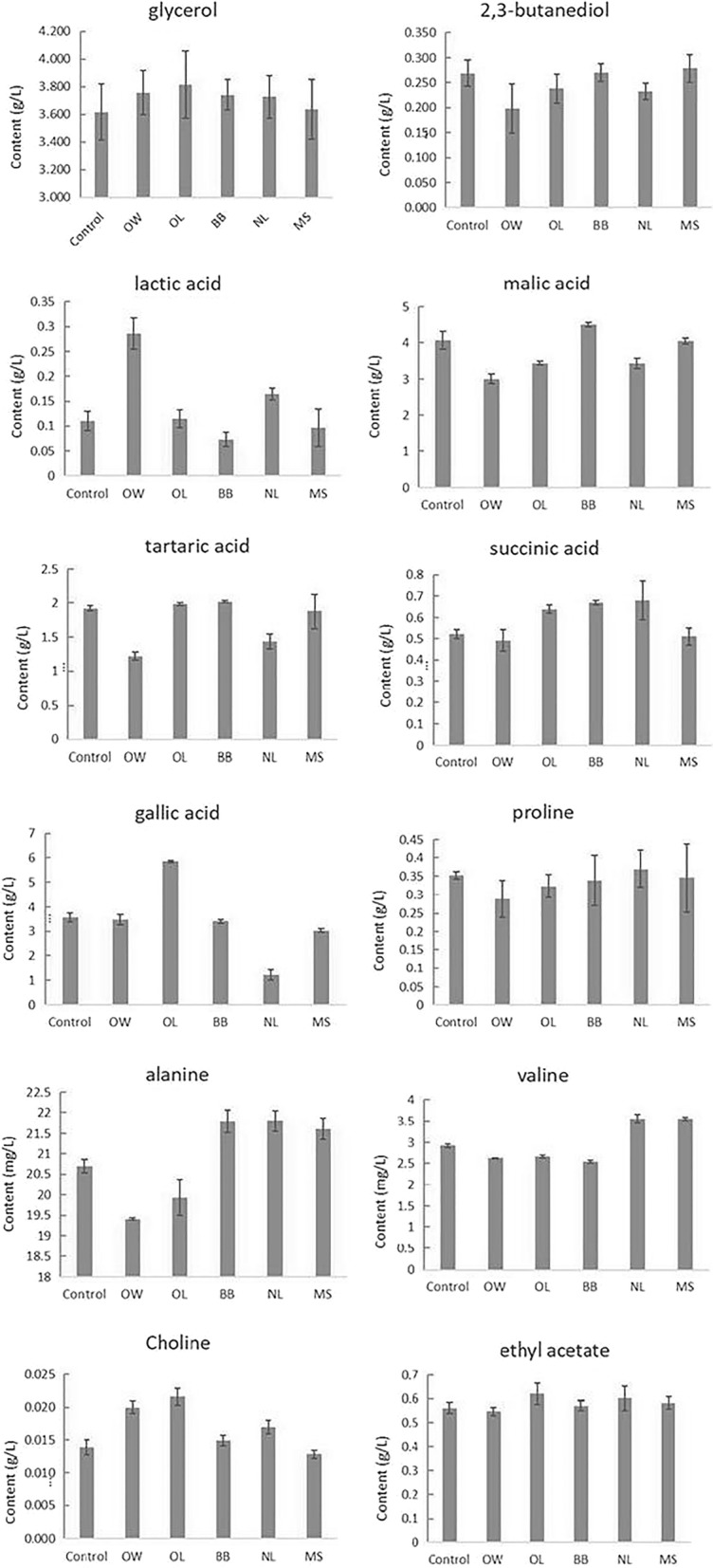
Content of the main metabolites in Chardonney dry white wine

^1^H NMR spectra of all wine samples is shown in the Fig. 2. It can be seen from the figure that most of the metabolites in Chardonnay dry white wine are concentrated in the range of 2.0–9.0 ppm, and the metabolites in the range of δ0.0–5.0 ppm are relatively dense. It was shown that there were many kinds of metabolites in this interval and the content was relatively high. Comparing with the chemical shift information of the wine metabolites based on the related references and database (Anastasiadi et al. 2009; Fotakis et al. 2013; Viggiani and Morelli 2008), combined with the NMR plot in the experiment, the metabolites in Chardonnay dry white wine was identified and the results were shown in Table 2. The mass concentration u (mg/L or g/L) of metabolites is shown in Fig. 3.

**Table 2:** ^1^H NMR assignment of metabolites in Chardonney dry white wine

Serial number	Metabolites	Nuclear magnetic resonance chemical shift
1	Valine	0.90(d, C4H_3_), 1.02(d, C5H_3_)
2	Ethanol	1.19(t, C2H_3_), 3.67(q, C1H_2_)
3	Acetic acid	2.07(s, C2H3)
4	2,3-butanediol	1.16(d, C1H_3_ + C4H3)
5	Succinic acid	2.68(s, C2H_2_ + C3H_2_)
6	Proline	2.00(m, γ-CH_2_), 2.07(m, β-CH), 2.35(m, β’-CH), 3.35(m, δ-CH), 3.40(m, δ-CH), 4.13(m, α-CH)
7	Ethyl acetate	1.26(t, C4H_3_), 4.18(q, C3H_2_)
8	Tartaric acid	4.57(s, C2H + C3H)
9	α-Glucose	5.25(d, αC1H)
10	β-Glucose	4.64(d, βC1H)
11	Lactic acid	1.42(d, C3H_3_), 4.29(m, C2H)
12	Gallic acid	7.15(s, C2H + C6H)
13	Glycerol	3.58 (q, C2H_2_), 3.67(q, C3H_2_), 3.81(m, C1H)
14	Citric acid	2.82(d, C2H_a_ + C4H_a_), 2.94(d, C2H_b_ + C4H_b_)
15	Choline	3.20(s, N-CH_3_), 3.52(t, αCH_2_), 4.09(t, βCH_2_)
16	α-d-Glucuronic acid	5.35(d, C1H)
17	Malic acid	2.78(dd, βCH_2_), 2.90(dd, β’CH_2_), 4.50(q, CH)
18	Alanine	1.52(d, βCH_3)_
19	d-Sucrose	5.41(d, C1H), 3.55(dd, C2H), 3.72(dd, C3H), 3.90(dd, C4H), 4.215(d, C1′H), 4.05(dd, C2′H), 3.88(dd, C3′H)
20	Tyrosine	6.86(d, C2H), 7.19(d, C3H)

The characters in brackets refer to peak information: s, singlet; d, doublet; t, triplet; q, quartet; dd, doublet of doublets; m, multiplet

Through the ^1^H NMR spectrum of wine samples, amino acids, organic acids, sugars and a small amount of phenolic substances were identified. These substances form the complex and unique flavors of the wine. In addition, most of the small molecule metabolites in the wine samples were the same, indicating that inactive yeasts did not change the type of metabolites in wine to a large extent, but significantly influenced their content. Therefore, ^1^H NMR can be used to analyze and identify the metabolites in the wine. However, small molecule metabolites may overlap at the peak position. Simply analyzing the nuclear magnetic spectrum cannot accurately describe the difference between the metabolites in the samples and the effect of inactive yeast on metabolites. Therefore, further use of pattern recognition method to analyze the data, extract the difference information of metabolites, and differentiate the relationship between the samples.

**Fig. 4:**
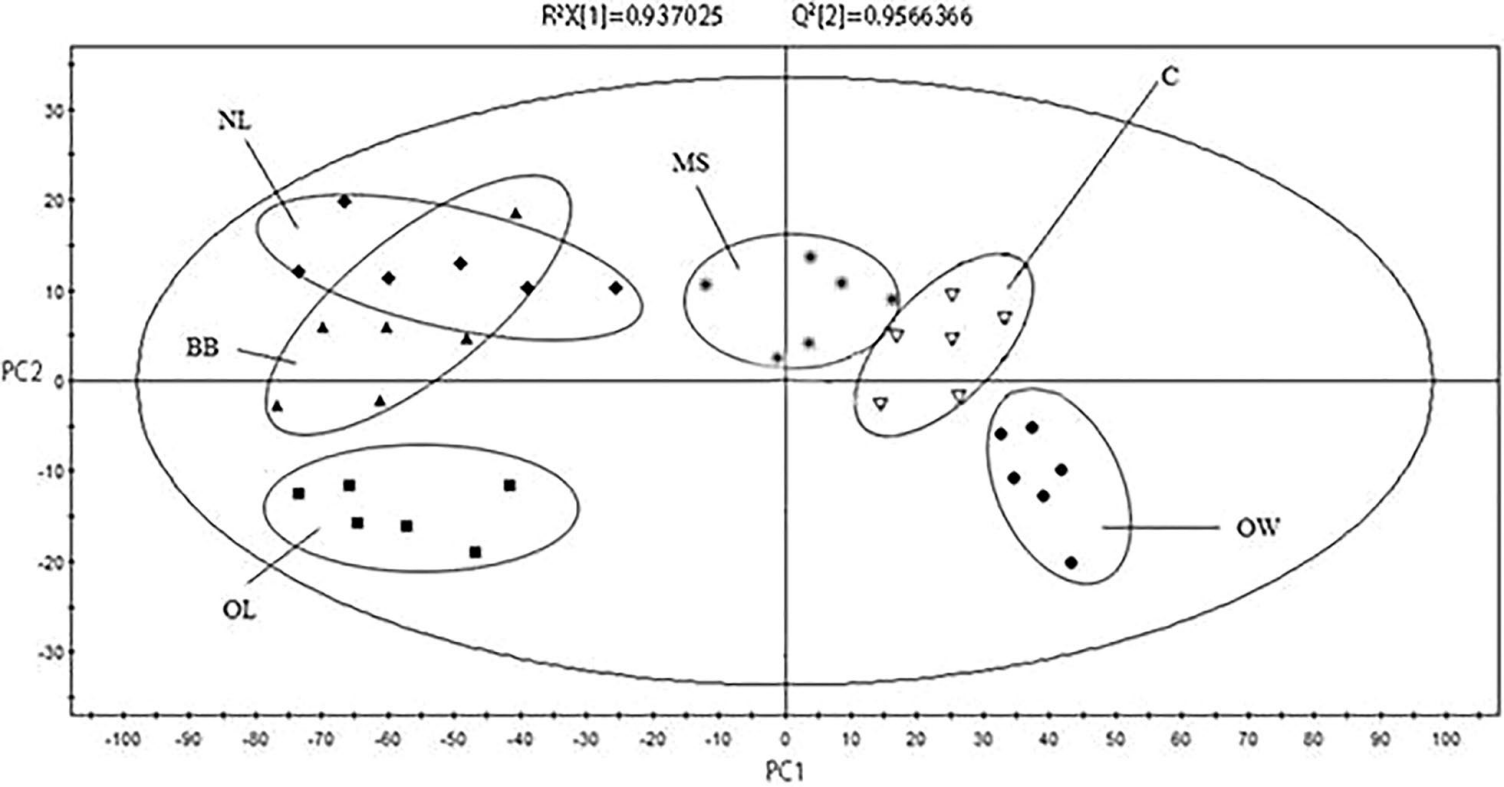
PCA scores plot (PC1/PC2) derived from the ^1^H NMR spectra of the Chardonney dry white wines with different inactive yeasts. *OW, OptiMUM-White^®^; OL, Opti-LEES; BB, Booster Blanc^®^; NL, Noblesse^®^; MS, Mannostab^®^; C, control)

PCA model was established based on the data obtained from the Chardonnay dry white wine produced in 2016 in Shacheng Manor Wine Co. Ltd., Hebei province, China. The PCA scores plot is shown in Fig. 4. The difference between Chardonnay wine and sample wine with the inactive yeast could be distinguished. Each yeast addition group also has varying degrees of dispersion. R^2^X = 0.937, Q^2^ = 0.957, indicating that the established PCA model is reliable.

**Fig. 5:**
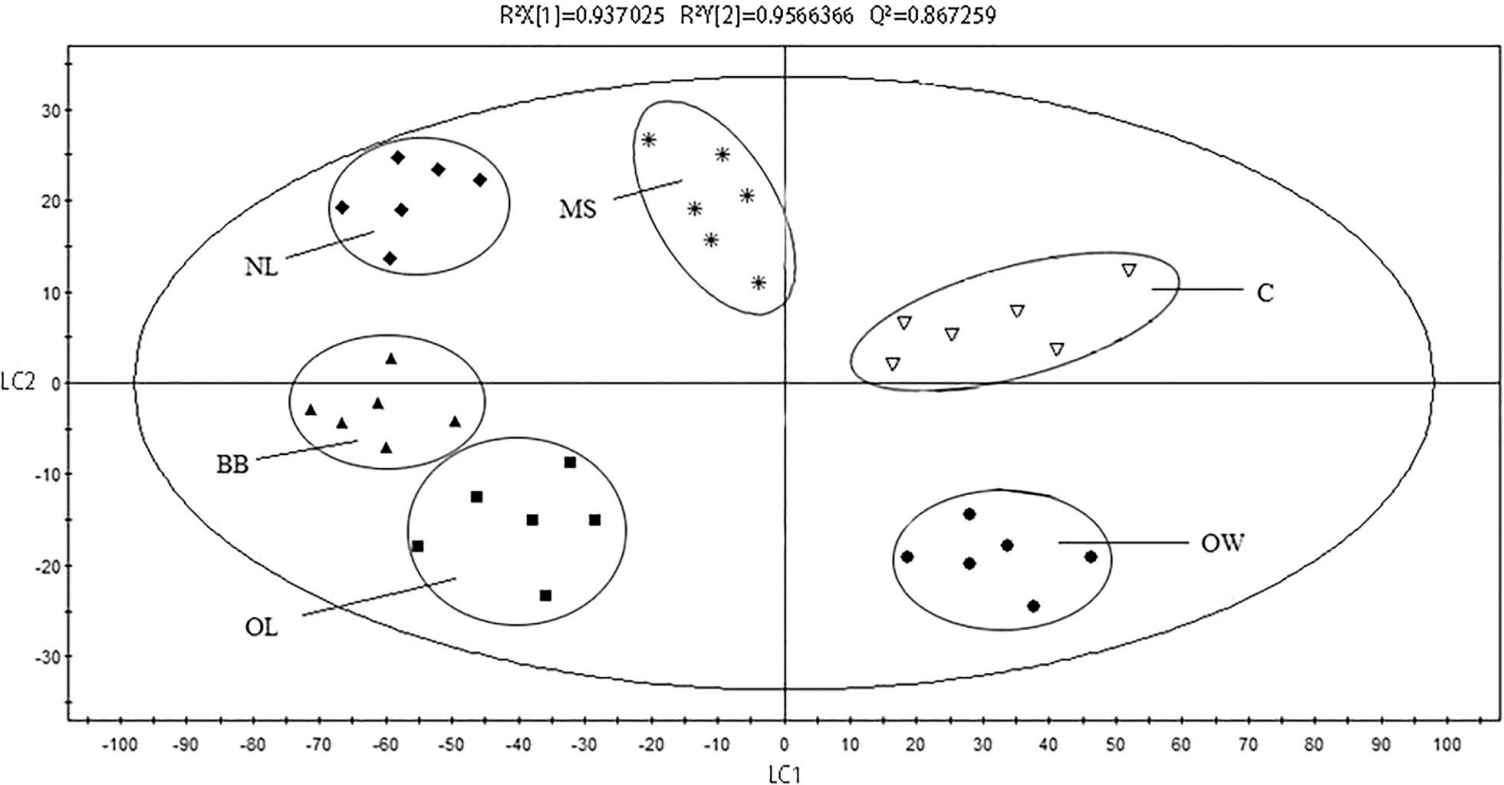
PLS-DA scores plot (LC1/LC2) derived from the ^1^H NMR spectra of the Chardonney dry white wines with different inactive yeasts. *OW, OptiMUM-White^®^; OL, Opti-LEES; BB, Booster Blanc^®^; NL, Noblesse^®^; MS, Mannostab^®^; C, control)

To further demonstrate the differences between wine samples, a PLS-DA model was established after the data was orthogonally corrected. PLS-DA scores plot is shown in Fig. 5. The R^2^X = 0.937, R^2^Y = 0.957, Q^2^ = 0.867, all of which were above 0.5, indicating that the model of the construction has a high quality. From the scores plot, the discrete relationship between the various wine samples could also be further revealed.

The results of the analysis showed that the white wine samples with five various inactive yeasts were more discrete, indicating that their metabolites were not identical.

**Fig. 6:**
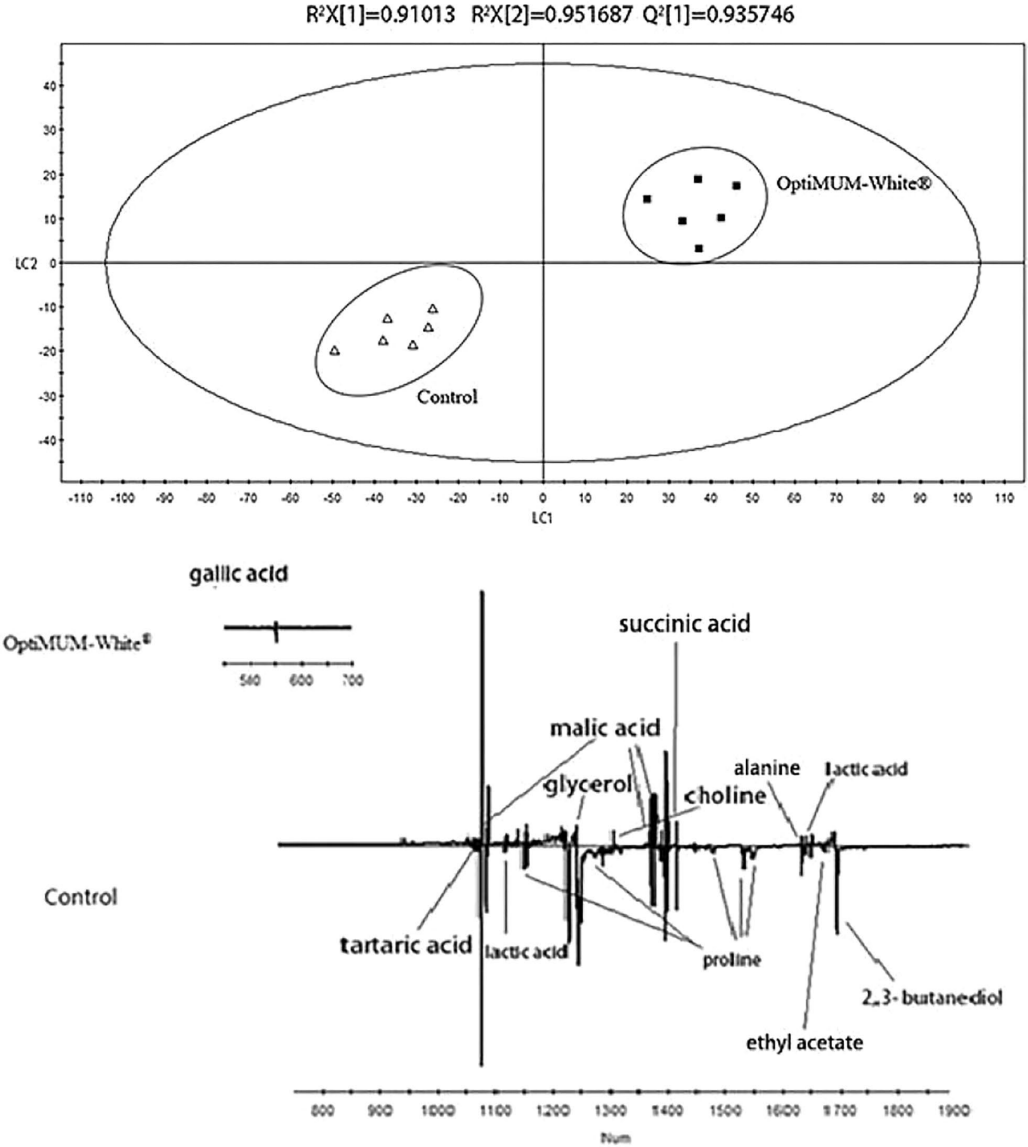
PLS-DA scores plot and loading plot derived from the ^1^H NMR spectra as pairwise comparison between Chardonney dry white wine and wine with OptiMUM-White^®^ (up: scores plot; down:loading plot)

The PLS-DA scores plot of the control group and white wines added with OptiMUM-White^®^ inactive yeast are shown in Fig. 6. As seen from the score plot, the two types of the wine can be clearly distinguished on the LC1 axis. The R^2^X = 0.910, R^2^Y = 0.952, and Q^2^ = 0.936, all of which are above 0.5, indicating that the model was highly reliable. Specific metabolic product information can be obtained from the load diagram. It was found that the wines added with OptiMUM-White^®^ inactive yeast had higher levels of lactate and choline than the control group of Chardonnay dry white wines. However, the content of 2,3-butanediol, ethyl acetate, proline, malic acid, tartaric acid, alanine, valine, succinic acid and glycerol was insignificantly lower than that of the original wine. As for the content of gallic acid, the differences were small.

**Fig. 7:**
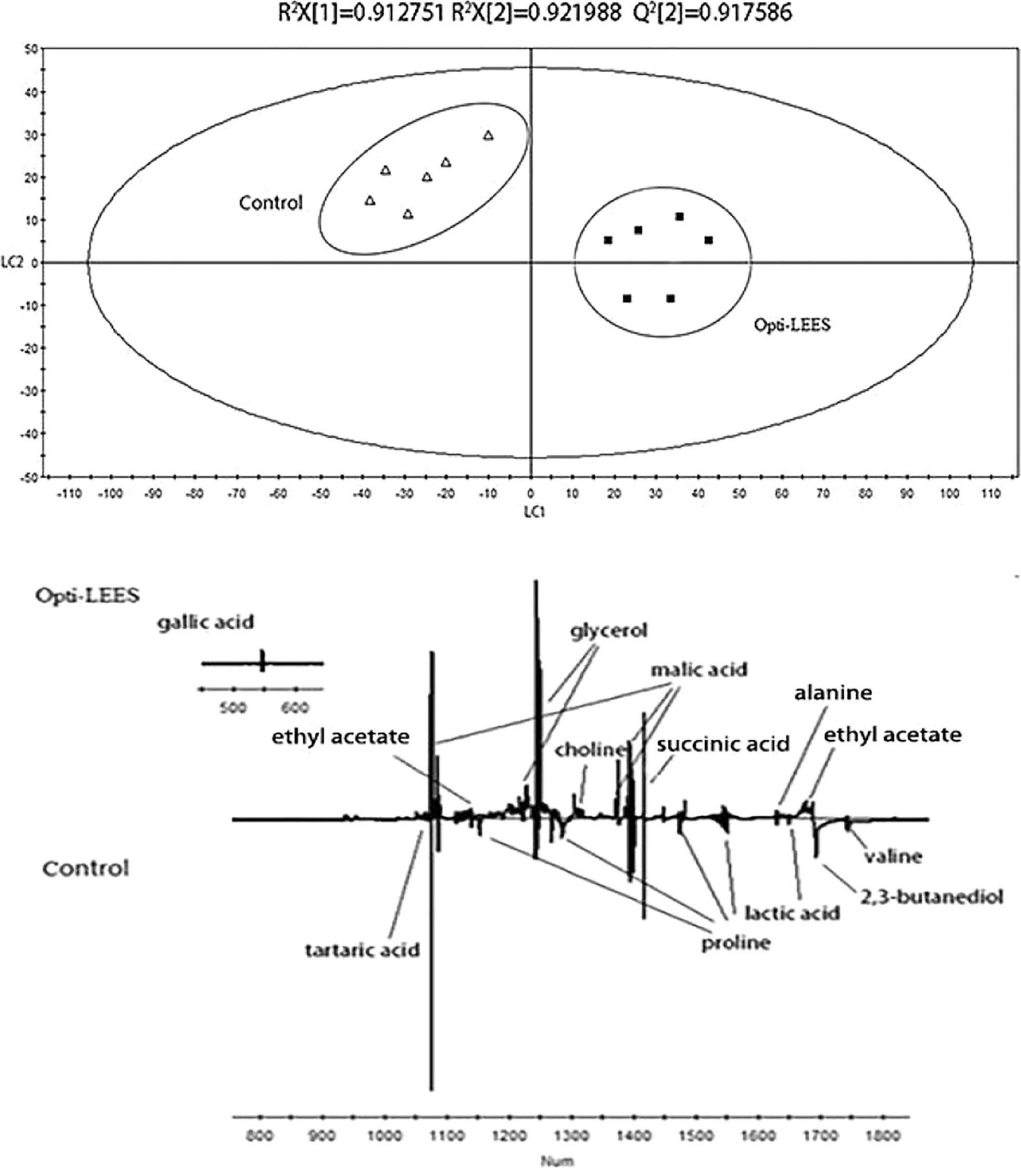
PLS-DA score plot and loading plot derived from the ^1^H NMR spectra as pairwise comparison between Chardonney dry white wine and wine with Opti-LEES (up: scores plot; down:loading plot)

The PLS-DA scores plot of the control group and white wines added with Opti-LEES inactive yeast is shown in Fig. 7. As seen from the score plot, the two types of wine can be clearly distinguished on the LC1 axis as well. The R^2^X = 0.913, R^2^Y = 0.922, Q^2^ = 0.918, all of which are above 0.5, indicating that the model has high reliability. Specific metabolic product information can be obtained from the loading plot. It was found that the wines added with Opti-LEES inactive yeast had higher levels of glycerol, ethyl acetate, gallic acid, succinic acid and choline. However, the content of proline, alanine, valine, 2,3-butanediol, and malic acid was relatively lower. The differences between the content of tartaric acid and lactic acid are insignificant.

**Fig. 8:**
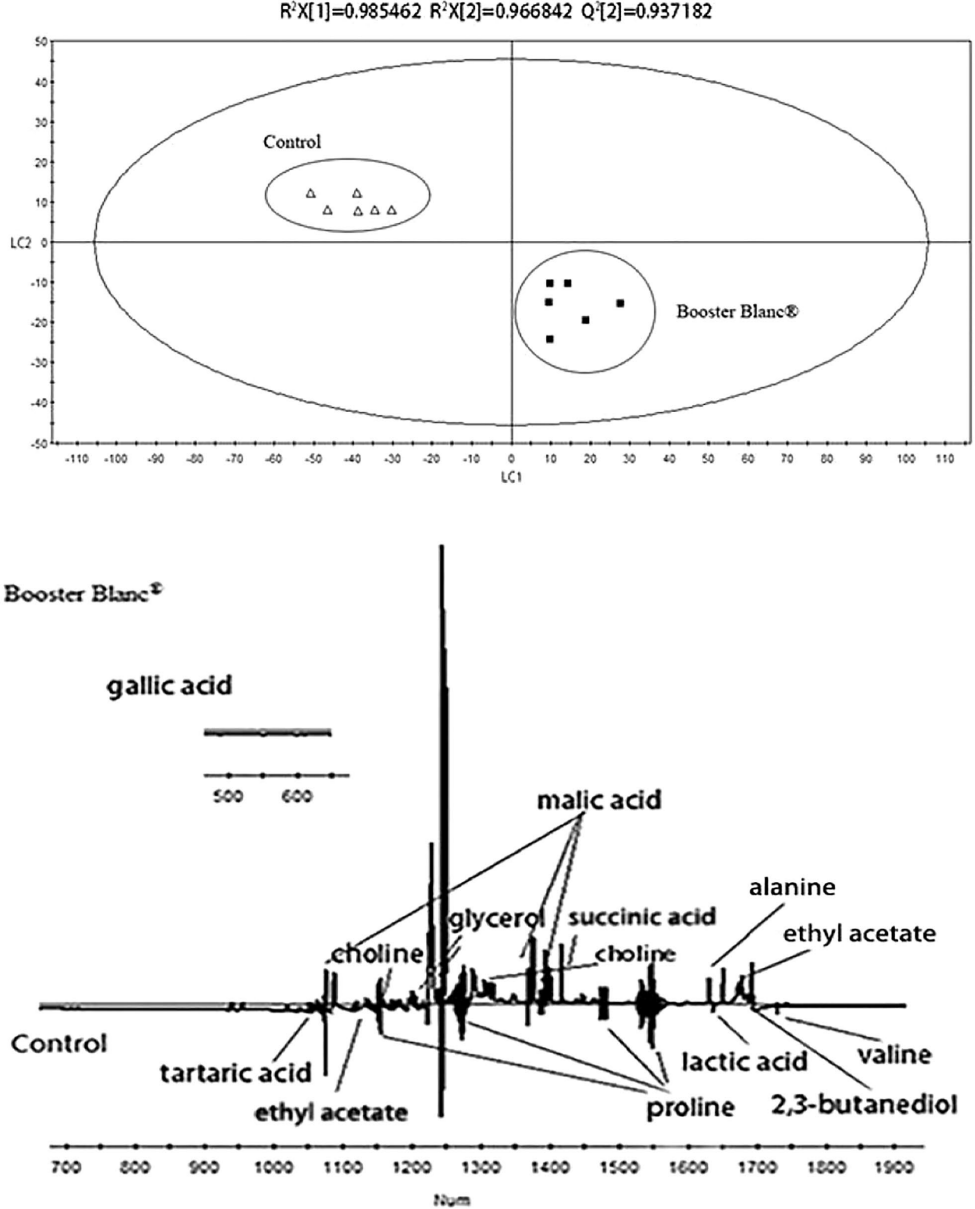
PLS-DA score plot and loading plot derived from the ^1^H NMR spectra as pairwise comparison between Chardonney dry white wine and wine with Booster Blanc^®^ (up: scores plot; down:loading plot)

The PLS-DA scores plot of the control group and white wines added with Booster Blanc^®^ inactive yeast is shown in Fig. 8. As seen from the score plot, the two types of wine can be clearly distinguished on the LC1 axis. The R^2^X = 0.985, R^2^Y = 0.967, Q^2^ = 0.937, all of which are above 0.5, indicating that the model has high reliability. Specific metabolic product information can be obtained from the loading plot. It was found that the wines added with Booster Blanc^®^ inactive yeast had higher levels of 2,3-butanediol, ethyl acetate, alanine, malic acid, succinic acid, tartaric acid, glycerol, choline. However, the content of proline, valine, lactic acid was relatively lower. There was little difference in the content of gallic acid.

**Fig. 9:**
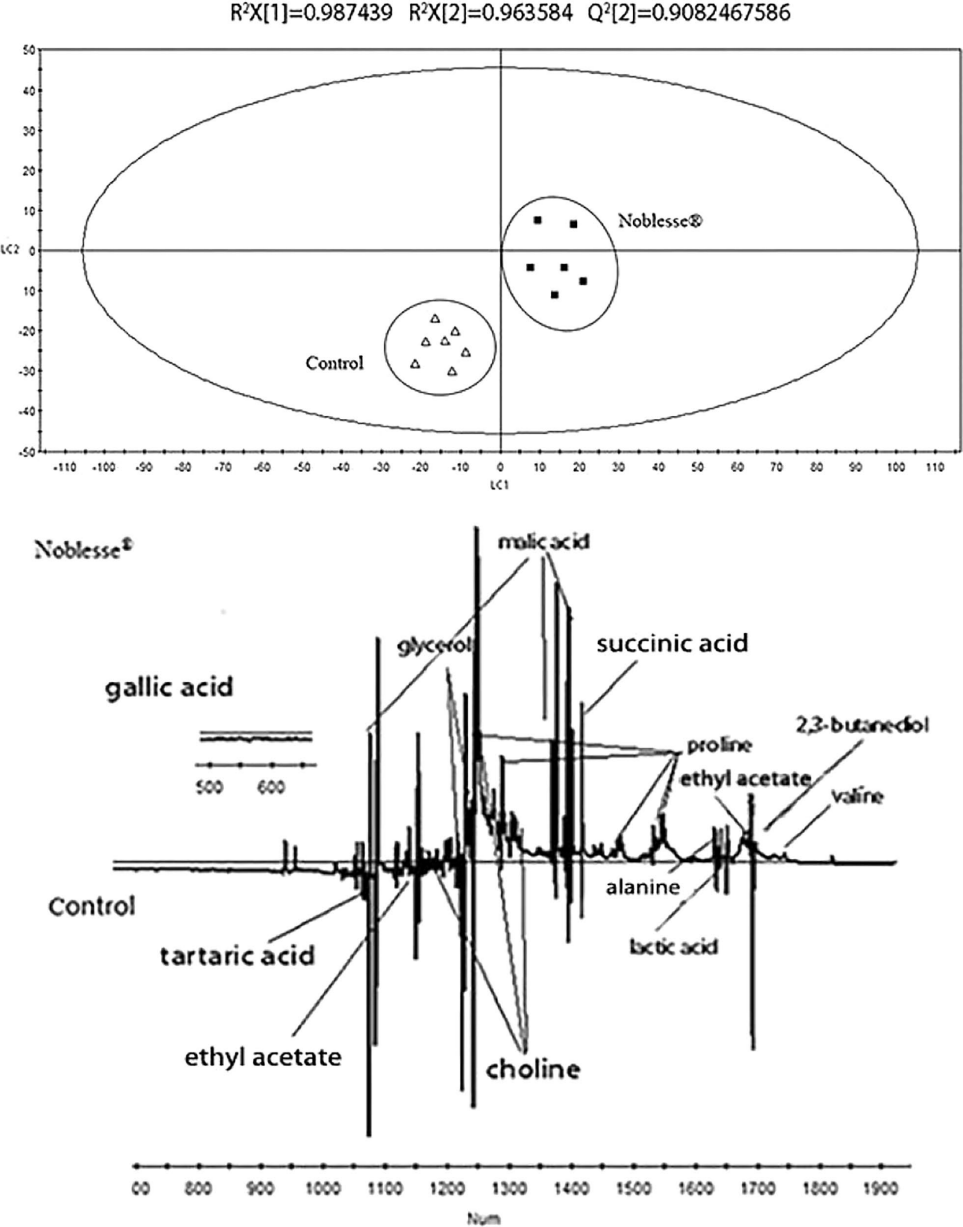
PLS-DA score plot and loading plot derived from the ^1^H NMR spectra as pairwise comparison between Chardonney dry white wine and wine with Noblesse^®^ (up: scores plot; down:loading plot)

The PLS-DA scores plot of the control group and white wines added with Noblesse^®^ inactive yeast is shown in Fig. 9. As from the score plot, the two types of wine can be clearly distinguished on the LC1 axis. The R^2^X = 0.987, R^2^Y = 0.964, Q^2^ = 0.825, all of which are above 0.5, indicating that the model has high reliability. Specific metabolic product information can be obtained from the loading plot. It was found that the wines added with Noblesse^®^ inactive yeast had higher levels of valine, alanine, proline, ethyl acetate, lactic acid, succinic acid, glycerol, choline. However, the content of malic acid, tartaric acid, 2,3-butanediol, gallic acid was relatively lower than that of the original wine.

**Fig. 10:**
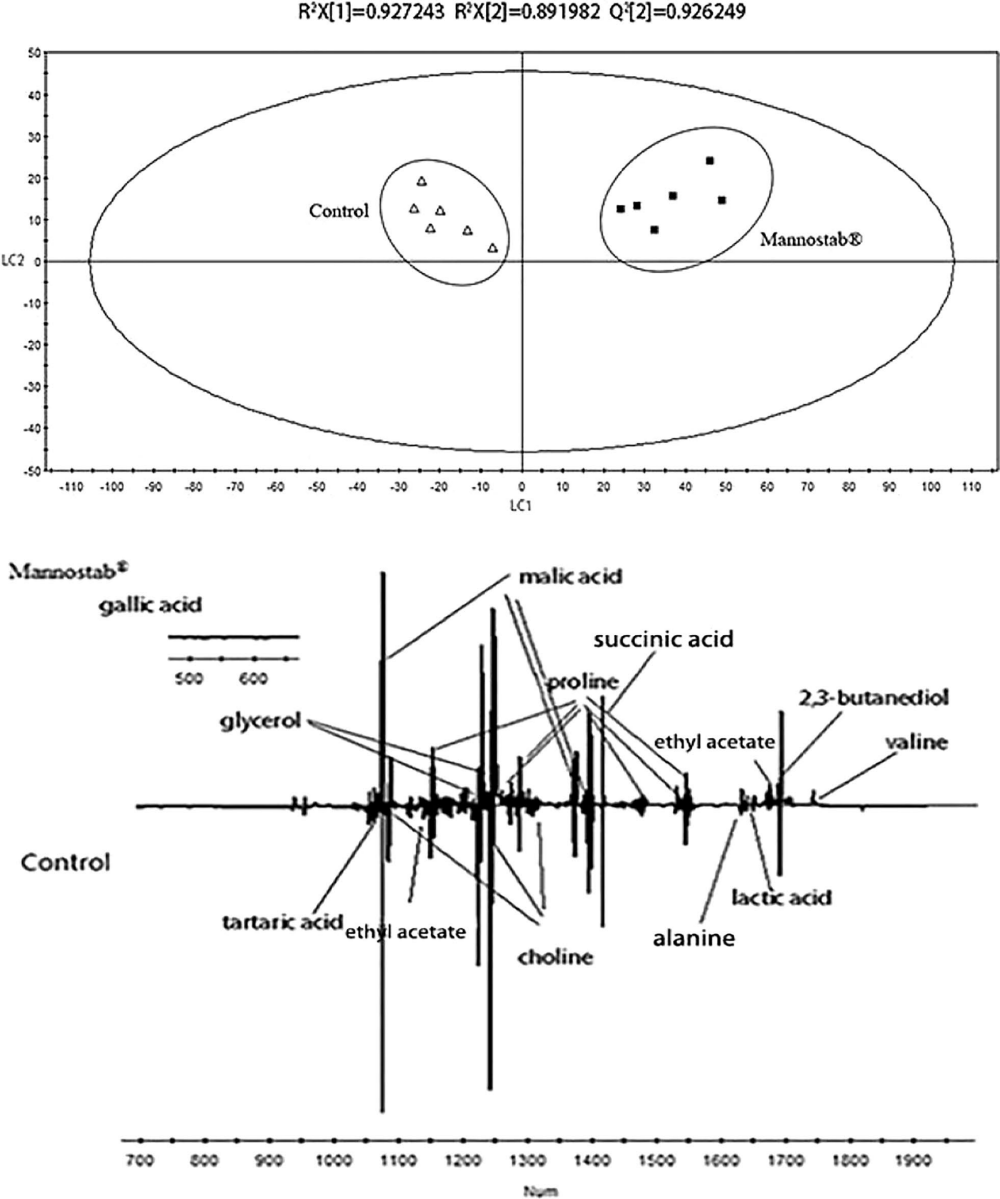
PLS-DA score plot and loading plot derived from the ^1^H NMR spectra as pairwise comparison between Chardonney dry white wine and wine with Mannostab^®^ (up: scores plot; down:loading plot)

The PLS-DA scores plot of the control group and white wines added with Mannostab^®^ inactive yeast is shown in Fig. 10. As seen from the score plot, the two types of wine can be clearly distinguished on the LC1 axis. The R^2^X = 0.927, R^2^Y = 0.892, Q^2^ = 0.926, all of which are above 0.5, indicating that the model has high reliability. Specific metabolic product information can be obtained from the loading plot. It was found that the wines added with Mannostab^®^inactive yeast had higher levels of valine, 2,3-butanediol, ethyl acetate and alanine. However, the content of malic acid, lactic acid, succinic acid, proline, tartaric acid, choline, glycerol, gallic acid was relatively lower than that of the original wine.

The content of polyols, organic acids, amino acids, choline and other metabolites in Chardonnay white wine from China in 2016 were determined by NMR, and the results are basically consistent with the loading plot.

In this study, the main metabolites of polyols are glycerol and 2,3-butanediol. Glycerol content in the wine samples with inactive yeast Opti-LEES, BoosterBlanc^®^, Noblesse^®^ was higher than the original wine. Moreover, the 2,3-butanediol content in Booster Blanc^®^ samples was higher than the original wine. This indicates that these three kinds of inactive yeast, especially Booster Blanc^®^, can increase the activity of yeast and improve the fermentation efficiency to a certain extent.

Malic acid in the wine with these two inactive yeasts including OptiMUM-White^®^ and Noblesse^®^ was lower than that of the original wine, while the content of lactic acid was relatively higher. The results showed that during the fermentation process the two strains of inactive yeast can improve the efficiency of malolactic acid fermentation. The contents of succinic acid and tartaric acid in wine samples with inactive yeast Booster Blanc^®^ and Biolees^®^, genesis lift were higher than those in the original wine.

Through NMR technique, the characteristic amino acids in the chardonnay dry white wine vinified in Shacheng Manor Wine Co. Ltd., Hebei province, China were mainly valine, proline and alanine. It was found amino acid content in the wines with the inactive yeast Noblesse^®^ and Mannostab^®^ was higher than that of the original wine. Especially in the wine with Noblesse^®^, the three amino acids were all increased to different levels. In a way, these inactive yeast could promote the autolysis of yeast cells and improve the metabolism of yeast.

The content of ethyl acetate in the wine with Opti-LEES, Booster Blanc^®^, Noblesse^®^, Mannostab^®^ was higher than that of the original wine, suggesting that the addition of inactive yeast could promote the fermentation of the wines, effectively improve the activity of yeast in the wine, and increase the fermentation efficiency. And it was found that the content of choline in the wine with OptiMUM-White^®^, Booster Blanc^®^, Opti-LEES and Noblesse^®^ was higher than that of the original dry white wine, indicating that these four kinds of inactive yeasts can promote the dissolution of choline to a certain extent in the fermentation process.

## Discussion

The main metabolites of the wine were determined by NMR spectra and chemical shift information. In order to distinguish the differences of metabolites between the wine with inactive yeast and the original wine, PCA and PLS-DA were used to analyse the characteristic metabolites of all samples and the original wine. The discrete trend and a similar relationship between the samples can be shown by PCA or PLS-DA. Their similar structural relationship is presented as Control > Optim-White^®^ > Noblesse^®^ > Opti-Lees > Boster Blanchc^®^ > Mannostab^®^.

This study found that there were significant difference between different inactive yeasts added to the wine and the original wine. The inactive yeast of wine had influence on the contents of polyols, organic acids, amino acids and other nutrients in the wine body. These metabolites can improve the nutrition and aroma of wine.

Glycerol and 2,3-butanediol are by-products of wine during alcoholic fermentation. Their content depends on the variety of wine grapes, yeast activity, fermentation temperature, pH, etc. In previous reports, Viggiani et al. confirmed the content of 2,3-butanediol was positively correlated with the content of alcohol and sugar, and the rate of alcohol fermentation was closely related to the activity of yeast flora (Viggiani and Morelli 2008). In this research, the main metabolites of organic acid are malic acid, lactic acid, tartaric acid and succinic acid. In the fermentation process, yeast can convert glucose to malic acid and lactobacillus can be further converted into pyruvate and lactic acid. The organic acids in wine can protect against microbial damage, but the over high content of organic acid can reduce the quality of wine. Excessive organic acid will lead to sour taste, hard, single style, lack of layers. Inactive yeast can promoted the acid reduction of the yeast, thereby modified the effect of the wine body style, highlighted the characteristics of the wine. In the process of wine brewing, part of the amino acids are released from the wine grape fruit. Moreover, the metabolism of yeast cells, the autolysis of cells and the enzymatic hydrolysis of small-molecule proteins in the wine also produce some amino acids. The wine was enrich amino acid for the inactive yeast can help to promote the autolysis of yeast. Choline is not only a water-soluble organic base in wine, but also a very nutritious substance (Zhu et al. 2018). It can promote brain development, improve memory, promote fat, transmethylation metabolism, and reduce serum cholesterol. Ethyl acetate is an important aroma substance in wine. Moderate amount of ethyl acetate can bring people slightly sweet fruit fragrance, making the wine body enrich and full-bodied layer. The content of ethyl acetate in wine is related to fermentation technology, grape variety and fermentation temperature.

In this study, Chardonnay dry white wine produced from China with the addition of inactive yeast and the original wine were detected and analyzed. The distinguished contribution of metabolites are glycerol, 2,3-butanediol, lactic acid, malic acid, tartaric acid, succinic acid, gallic acid, proline, alanine, valine, ethyl acetate and choline. Through these metabolites we can differentiate all samples. By pattern recognition of PCA and PLS-DA, the wine samples can be distinguished. It indicated that the addition of inactive yeasts can change the contents and types of metabolites in the wine. It provides a theoretical basis for the optimization and the method for improving the nutrition and quality of dry white wine brewing process. The results showed that the metabolites of the wine samples with the addition of inactive yeast were different from the original wine.

## Data Availability

All raw data are available at the corresponding author.

## Abbreviations

NMR: nuclear magnetic resonance spectroscopy
PCA: principle component analysis
PLS-DA: partial least squares discrimination analysis
LC–MS: liquid chromatography–mass spectrometry
GC–MS: gas chromatography–mass spectrometry
DSS: 4,4-dimethyl-4-silapentane-1-sulfonic acid
